# Supplementary material for: Overexpression of PtrMYB121 Positively Regulates the Formation of Secondary Cell Wall in Arabidopsis thaliana
Source: Int J Mol Sci. 2020 Oct 19;21(20):7734. doi: 10.3390/ijms21207734 (PMC7589094; doi:10.3390/ijms21207734)
Supplement: Supplementary file 1 [file ijms-21-07734-s001.pdf]

Overexpression of *PtrMYB121* Positively Regulates the Formation of Secondary Cell Wall in *Arabidopsis thaliana*

Ying Liu [1], Jiayin Man [1], Yinghao Wang [1], Chao Yuan [1], Yuyu Shi [1], Bobin Liu [2], Xia Hu [1,3], Songqing Wu [3], Taoxiang Zhang [1,*] and Chunlan Lian [4,*]

1 International Joint Laboratory of Forest Symbiology, College of Forestry, Fujian Agriculture and Forestry University, Fuzhou 350002, China; yingliu1112@163.com (Y.L.); jiayinmanjiayin@163.com (J.M.); 18438616077@163.com (Y.W.); Yuanchao507@163.com (C.Y.); yuevan2014@163.com (Y.S.); lake-autumn@163.com (X.H.)

2 Fujian Colleges and Universities Engineering Research Institute of Conservation and Utilization of Natural Bioresources, College of Forestry, Fujian Agriculture and Forestry University, Fuzhou 350002, China; liubobin@fafu.edu.cn

3 Key Laboratory of Integrated Pest Management in Ecological Forests, Fujian Province University, Fujian Agriculture and Forestry University, Fuzhou 350002, China; dabinyang@126.com

4 Asian Natural Environmental Science Center, The University of Tokyo, 1-1-8 Midori-cho, Nishitokyo, Tokyo 188-0002, Japan

* Correspondence: xsnzheda2009@163.com (T.Z.); lian@anesc.u-tokyo.ac.jp (C.L.)

Supplementary Materials:

Table S1. The list of primers used in this study.

Name	Forward Primer Sequence (5′–3′)	Reverse Primer Sequence (5′–3′)
**Primers for CDS amplification of PtrMYB121**		
PtrMYB121	ATGGGGAGGCACTCTTGTTGCT	CTAAACATATCCATAAGATGCTGTG-
**Primers for identify of PtrMYB121-overexpression transgenic lines**		
PtrMYB121(fragment)	TCTGAGGTTGAAGATGGAGAAG	GTCCTCCTGTTGGCTTCC
**Primers for Transient Expression Assay（LUC）**		
ProAtCAD5	CATGGATAAGTATCAACTGTTCTC-	CCTCTCTGCCTCCATTATTCCCAT'
ProAtCOMT	GTATTCGTCATGCTATGACGACG	GTGTCTCTGCCGTTGAACCCAT
ProAtCCOAOMT1	CATACTCAACCATTCGCATTACGC-	GCTTCTGTTGTTGTCGTCGCCAT
ProAt4CL2	AGGCCTCGTTGGACTTTCGCTCTC	CTATCACATCTTGTGTCGTCAT
ProAtCesA7	CATCATAGCTCGACCTCTGATGAT	ACAAGACCGGCGCTAGCTTCCAT
ProAtCesA8	CAAGCTGATAGTATAGGACTGG	GGAGACCTAGACTCCATCA
**Primers for qRT-PCR**		
AtUBQ	GTAAGCAGCTTGAAGATGGAAG	ATTGTTTTACCCGTCAGAGTCT
AtCAD5	CACCAAAAGTTTGTGGTCAAGA	CTGGTTGTTTCAGACCAAAGTG
AtC3H1	AGATGTTGACATGAAGGGTCAT	AATTGATACCAAGTTGTGCACC
AtCOMT	GGCTTTAAAATCCGCCTTAGAG	CAGGATTTTTGGTCGGAAGTTT
AtC4H	GAAGTCTTTAATCGCCGTCTTC	TGAGATCATCTCCGACTTGAAG
AtCCOAOMT1	GTTGATGCTGACAAAGACAACT	GCCTTGTTAAGCTCAAGAACAA
AtF5H1	GCACACTCAAAGAAACCCTAAG	AGAAACCGTCGATACTAGTGTC
AtPAL3	GATCCGCTTCAGAAACCTAAAC	ACATCGATCAAAGGGTTATCGT
At4CL2	CGAAACTCATCGTCACTCAATC	AATCTTCTCCGGTATTGAGTCC
AtCesA7	GACCTATCTAGATCGGCTTTCC	ACTGACAAAGACATCTACAGGG
AtCesA8	GCTTTCGGTTGTGATTCCTATC	CACAGATGTTAGCCATAAACCG
AtIRX9	AAAGTGACACCAAGAGGACTAG	CGAGTGTTTTTCCACGACTATC
AtIRX14	CTCTTGATTCTTCCGTTGATGG	CGAAAGCCGAGAACTAGACTAA
PtrMYB121	ATGGAGCCTCAATTTAGACGAA	AAGAGAAAAATCTATGGCACGC
PtrMYB55	AAAATTCAAGTGGTATGTGGCC	TCTAAATTGAGGCTCCCTGTAC
PtrActin	AAGGTTGTTGCACCACCAGA	AACACACAGTCCATCACCGC

Table S2. Physiological indexes in WT and PtrMYB121-overexpressing *A. thaliana* (40-day-old plants).

	WT		PtrMYB121		
	L1	L2	L1	L4	L6
Trichome density (/25 mm^2)	23.62±3.31	24.29±3.60	22.52±3.18	24.73±4.04	23.88±3.97
Plant height (cm)	18.96±0.23	17.95±0.21	19.17±0.36	18.36±0.19	18.54±0.28
Plant diameter (cm)	9.47±0.11	9.18±0.14	8.95±0.09	9.53±0.19	9.21±0.11
Leaf area/ per Rosette leaf (cm^2)	7.15±0.14	6.91±0.12	7.09±0.16	6.95±0.08	7.31±0.23

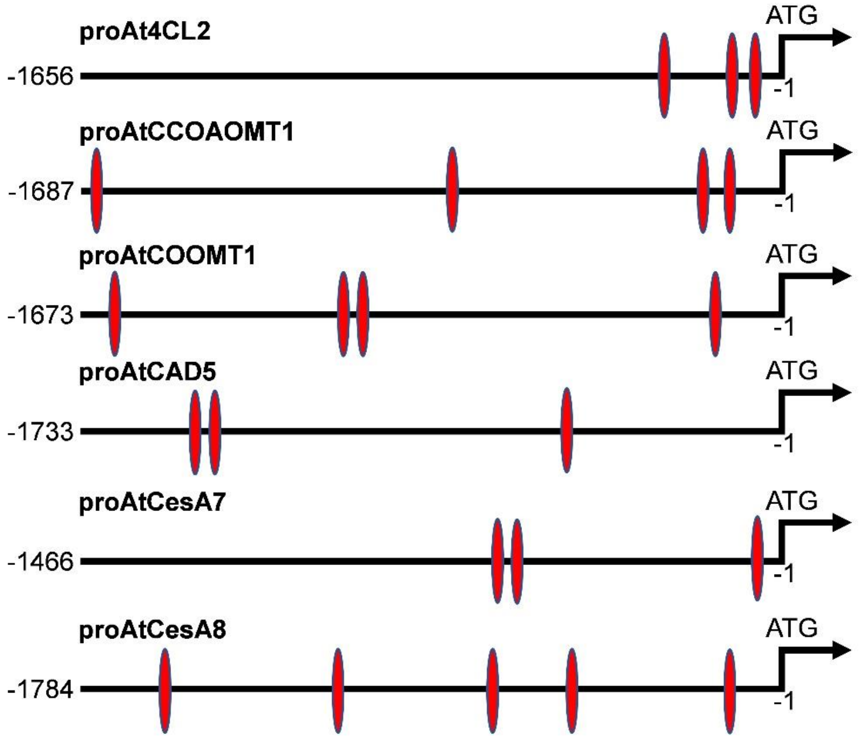

Figure S1. Cis-element analysis of the promoters of lignin and cellulose biosynthetic genes. The promoter sequences of *A. thaliana* lignin and cellulose biosynthetic genes, including *4CL2*, *CCOAOMT1*, *COMT1*, *CAD5*, *CesA7* and *CesA8*. Distribution of secondary wall MYB-responsive elements ACC(A/T)A(A/C)(T/C) in the promoter regions was marked with rex box. The numbers indicate the length of promoter sequences.
